# Virus-Free Micro-Corm Induction and the Mechanism of Corm Development in Taro

**DOI:** 10.3390/ijms26083740

**Published:** 2025-04-16

**Authors:** Shenglin Wang, Yao Xiao, Zihao Li, Tao Liu, Jiarui Cui, Bicong Li, Qianglong Zhu, Sha Luo, Nan Shan, Jingyu Sun, Yingjin Huang, Qinghong Zhou

**Affiliations:** Jiangxi Province Key Laboratory of Vegetable Cultivation and Utilization, Jiangxi Agricultural University, Nanchang 330045, China

**Keywords:** *Colocasia esculenta* (L.) Schott, virus-free micro-corm induction, corm development, abscisic acid, starch

## Abstract

Taro (*Colocasia esculenta* (L.) Schott) is the fifth largest rhizome crop, and it is widely distributed in tropical and subtropical areas in the world. Vegetative propagation with virus-infected corms can lead to cultivar degradation, yield decline, and quality deterioration. In this study, the shoot apical meristems excised from taro corms infected with dasheen mosaic virus, which belongs to the genus *Potyvirus* in the family *Potyviridae*, were cultured and treated with exogenous abscisic acid and high sucrose concentrations to induce micro-corm formation. Subsequently, candidate genes involved in micro-corm expansion were screened via transcriptome sequencing analysis. The results revealed that the shoot apical meristems could grow into adventitious shoots on the medium 1 mg/L 6-benzylaminopurine + 0.3 mg/L 1-naphthaleneacetic acid, and reverse transcription–polymerase chain reaction detection indicated that dasheen mosaic virus had been successfully eliminated from the test-tube plantlets. Moreover, 8% sucrose or 3% sucrose + 5 μM abscisic acid likewise induced taro corm formation, and genes related to cell division and the cell cycle, as well as starch and sucrose metabolism pathways, were significantly enriched during taro corm expansion. Furthermore, the cyclin-dependent kinases genes, cell cycle protein kinase subunit genes, and cyclin B2 genes, which are related to cell division and the cell cycle, were upregulated with abscisic acid treatment on the 3rd day. The sucrose synthase genes, β-amylase genes, glycogen branching enzyme genes, and soluble starch synthase genes, which are related to starch and sucrose metabolism, were upregulated on the 15th day, indicating that cell division largely occurs during taro corm formation, whereas carbohydrates are synthesized during taro corm expansion.

## 1. Introduction

Taro (*Colocasia esculenta* (L.) Schott) is an important starch crop and vegetable that belongs to the *Araceae* family; it grows naturally in India and Southeast Asia and is widely cultivated in Asia and East Africa [1]. In 2021, the global area of taro planted was approximately 1.87 million hectares, and production reached 14.31 million tons (Food and agriculture organization of the United Nations (FAO), 2021, available at http://www.fao.org/faostat/en/#data/QC, accessed on 1 February 2024). Taro is not only a great source of starch but also rich in bioactive substances such as polysaccharides, proteins, flavonoids, and saponins [2,3].

As an asexual reproduction crop, the taro corms as seeds accumulate many viruses with constant reproduction of their somatic cells, such as dasheen mosaic virus (DsMV), cucumber mosaic virus (CMV), and taro bacilliform virus (TaBV), which leads to cultivar degradation and a decline in yield and quality [4]. In the past, shoot apical meristem culture was employed to eliminate DsMV and other phytopathogens in caladium, taro, and cocoyam [5]. In later studies, thermotherapy was applied by exposing taro seeds to 60 °C for 120 min or 55 °C for 120 min; although the regenerated plants were free of all viral disease symptoms, this treatment significantly reduced seed germination rates [6]. To improve efficacy, a combined method of shoot apical meristem culture and thermotherapy was developed, proving to be an effective strategy for eradicating taro viruses, such as DsMV [7,8]. Furthermore, shoot-tip cryotherapy was effective in eliminating DsMV and completely eradicating CMV in red bud taro [9]. In other crops such as potato and apple, the combination of thermotherapy and/or chemotherapy with cryotherapy has been effective in eradicating viruses to ensure the production and supply of high-health planting material to the industry [10,11]. Virus-free healthy materials can maximally preserve the desirable genetic traits of germplasm, fully highlighting its potential for high yield and superior quality. This is highly important for the preservation of taro germplasm resources and the breeding of new varieties.

Tuber formation and expansion are important factors in determining yield and quality in root and tuber crops such as potato, yam, and taro [12,13]. Plant tuber formation is a complex biological process involving many genes of multiple metabolic pathways. For example, gibberellin inhibits plant tuber formation, and the overexpression of the gibberellin 20-oxidase gene 1 (*GA20ox1*) leads to internode elongation and delayed tuber formation [14]. Abscisic acid (ABA) increases the contents of sucrose and starch in Gladiolus bulbs by increasing the expression of the sucrose synthase gene (*GhSUS2*) and positively regulates bulb development [15]. Previously, we revealed the mechanisms of taro corm formation and development, and reported that the ABA content remained high during in vitro virus-free taro corm expansion [16]. In addition, auxin [17] and cytokinin [18] also affect plant tuber formation, and phytochrome F (*StPHYF*) and phytochrome B (*StPHYB*) form dimers to inhibit the formation of microtubers under long-term sunshine in potato [19].

Exogenous sucrose is an important and commonly used sugar source in plant tissue culture and plays a crucial role during the expansion of tubers [20], which primarily facilitates the bulking of microtubers and regulates tuber metabolism at both the transcriptional and posttranscriptional levels [21,22]. The microtubers are produced in vitro by subculturing single-stem node cuttings on a Murashige and Skoog (MS) medium supplemented with 8% sucrose [23,24]. During the bulking phase, the starch content in storage roots increases, whereas the sucrose content decreases in sweet potato [25]. Additionally, sucrose is involved in various metabolic pathways, activating genes related to tuber growth, thereby directly affecting the formation and development of tubers [26,27,28].

Here, we obtained virus-free test-tube corms, which are easier to transport and store than the test-tube plantlets. The effects of exogenous ABA and sucrose on taro corm formation and expansion are also clarified. Additionally, genes related to starch and sucrose metabolism during swelling are also identified. These results provide important information for the regulation of taro corm formation and development. The RNA-seq data also provide important resources for future functional studies and shed light on the genetic control of taro.

## 2. Results

### 2.1. Establishing a Rapid Propagation System for Tissue Culture and Obtaining Virus-Free Taro Plantlets

**Figure 1:**
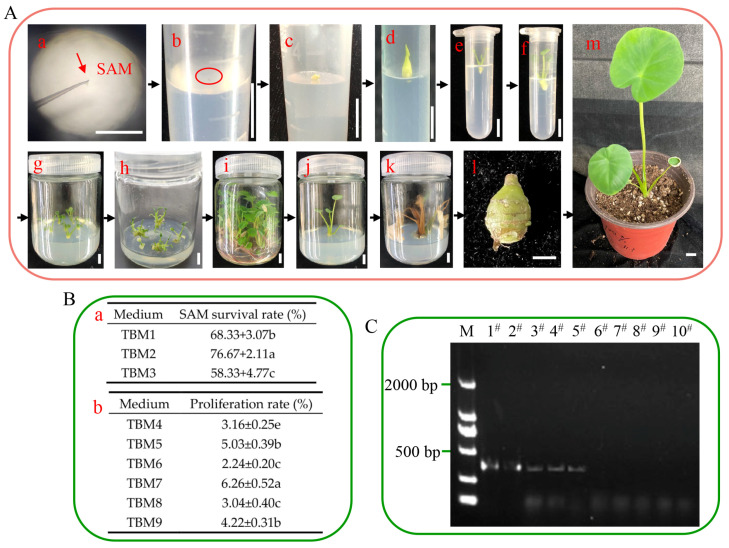
(**A**) Establishment of tissue culture and rapid propagation of taro and virus detected. To obtain the shoot apical meristems, the taro stem tips were unfolded under an anatomical microscope (**a**), the shoot apical meristems grew into 1.5 cm adventitious shoots in the TBM2 medium ((**b**): 0 d; (**c**): 10 d; (**d**): 30 d; (**e**): 70 d; (**f**): 90 d), proliferation of adventitious shoots in the TBM7 medium was observed ((**g**): 0 d; (**h**): 15 d; (**i**): 45 d), induction of test-tube taro in the TBM14 medium occurred ((**j**): 0 d; (**k**): 90 d), the test-tube taro corm grew (**l**), and the taro corm was subsequently grown into taro plants (**m**). Bar = 1 cm. (**B**) Tissue culture medium. Data are represented as the mean ± SD of three biological replicates. Different letters indicate a significant difference at *p* < 0.05 by Duncan’s test. The statistics of the shoot apical meristem survival rate in different media in 30 d (**a**) and the statistics of virus-free shoot proliferation rate in different media in 30 d (**b**). (**C**) RT-PCR assays were used to detect taro virus. M, marker Wells 1^#^–10^#^ were used to detect the DsMV (wells 1^#^–5^#^ were field plants, and wells 6^#^–10^#^ were test-tube shoots).

To obtain virus-free taro plantlets quickly in vitro, the shoot apical meristems were extracted from the tip of the taro stem (Figure 1A(a)). These shoot apical meristems were then inoculated into different media to induce growth in vitro. The results indicated that the survival rate of the shoot apical meristem was 76.67% in the TBM2 medium (Figure 1B(a)). The shoot apical meristem expanded and generated small calluses within 10 d, after which the tip elongated and gradually turned green while the leaf primordium unfolded in 30 d. Ultimately, small test-tube shoots measuring 1 cm were obtained after 70 d in the TBM2 medium (Figure 1A(b–f)). Next, we detected the virus in the field-grown plants and shoots via reverse transcription–polymerase chain reaction (RT-PCR) and detected DsMV in the field-grown plants but not in the test-grown shoots (Figure 1C). These results revealed that DsMV can be removed by shoot apical meristem culture in 93.3% of the in vitro generated plants. To obtain many virus-free taro plantlets, we transferred the virus-free shoots confirmed by RT-PCR to different media for proliferation (Figure 1A(g–i)) and found that the maximum proliferation rate was 6.26 in the TBM7 medium (Figure 1B(b)). We further transplanted virus-free plantlets into the TBM14 medium to induce test-tube taro corm, and we obtained a test-tube taro corm in 50 d (Figure 1A(j–l)). Later, the test-tube taro corm grew into a complete plant (Figure 1A(m)).

### 2.2. Synergistic Effects of ABA and Sucrose on Taro Corm Expansion

**Figure 2:**
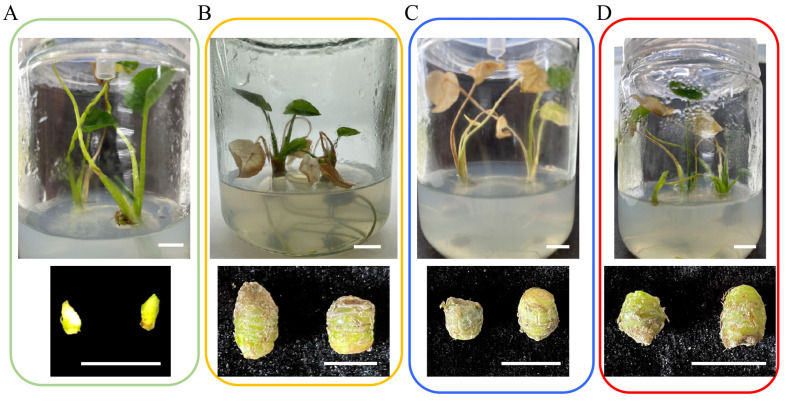
Expansion of taro corms induced by ABA and high sucrose concentrations for test-tube plantlets and taro corms in different media after 90 d. The top row shows the test-tube plantlets, and the bottom row shows the taro corms. (**A**) The TBM10 medium was unable to induce taro corm. (**B**) The TBM14 medium induced taro corms. (**C**) The TBM15 medium induced small taro corms, but the test-tube plantlets withered. (**D**) The TBM16 medium induced small taro corms. Bar = 1 cm.

We analyzed the effects of sucrose and ABA on the expansion of taro corms and found that taro corms were unable to be induced in the TBM10 medium (Figure 2A). However, 0.4 g of taro corms was induced in the TBM11 medium, and 0.28 g of taro corms was induced in the TBM12 medium (Table 1). These findings suggest that taro corms can be induced by ABA. Compared with the TBM10 medium, taro corms can be induced in the TBM13 medium, with a corm weight of 0.5 g, a transverse stem of 8.79 mm, and a longitudinal diameter of 11.85 mm (Figure 2B). These findings suggest that taro corms can be induced by high concentrations of sucrose.

Interestingly, the TBM14 medium induced the maximum weight of the taro corm, with a weight of 0.62 g, a transverse stem measuring 9.72 mm, and a longitudinal diameter measuring 11.98 mm (Figure 2B). On the other hand, the TBM15 medium only induced a taro corm weight of 0.26 g, and the test-tube plantlets withered (Figure 2C), suggesting that high ABA concentrations inhibited the formation of taro corms. Furthermore, compared with the TBM13 medium, the TBM16 medium induced smaller taro corms, with a weight of only 0.3 g, a transverse stem measuring 6.65 mm, and a longitudinal diameter measuring 8.01 mm (Figure 2D).

### 2.3. Analysis of Transcriptome Sequencing Data

To further analyze the molecular mechanism of ABA-induced taro corm expansion, test-tube plantlets that were 5 cm tall were transferred into the TBM13 medium or the TBM14 medium to induce taro corm growth. RNA-seq analysis of taro samples in contact with the medium was performed on the 3rd and 15th days. A total of 59.36 Gb of clean read data were generated from nine libraries, with average Q20 and Q30 values of 97.09% and 88.96%, respectively. The clean reads were mapped to the taro genome, and the unique reads accounted for 82.62–85.45% (Table S1).

**Figure 3:**
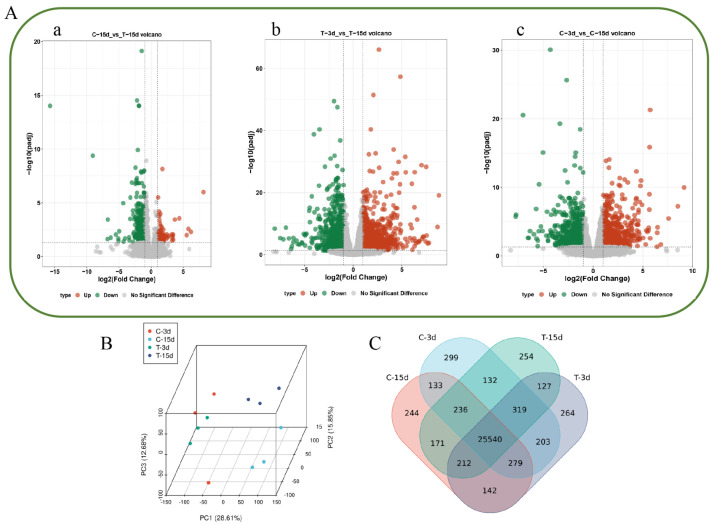
Transcriptome analysis of taro corm expansion. (**A**) Volcano plot of DEGs: C15 vs. T15 (**a**), T3 vs. T15 (**b**), and C3 vs. T15 (**c**). (**B**) PCA of gene expression in various taro samples. (**C**) Venn diagram illustrating gene expression analysis in different taro samples.

To identify differentially expressed genes (DEGs) among the ABA treatment groups, pairwise differential expression analysis on different treatment groups was performed. A total of 334 DEGs were identified in C15 vs. T15, of which 69 were more highly expressed in T15 and 265 were more highly expressed in C15 (Figure 3A(a)). A total of 2472 DEGs were identified in T3 vs. T15, of which 1098 DEGs were highly expressed in T15 and 1374 DEGs were more particularly expressed in T3 (Figure 3A(b)). A total of 1692 DEGs were identified in C3 vs. C15, of which 697 DEGs were more highly expressed in C3 and 995 DEGs were more highly expressed in C15 (Figure 3A(c)).

Principal component analysis (PCA) of the transcriptome datasets revealed that the 12 samples formed four groups: Group 1 (C-3d), Group 2 (T-3d), Group 3 (C-15d), and Group 4 (T-15d) (Figure 3B). Transcript abundances were determined by FPKM values, and a total of 58884 transcripts (FPKM > 0.01) were found among the 12 samples. Among them, 46184 genes were widely expressed among the 12 samples. A further 299, 244, 264, and 254 genes were specifically expressed at C-3d, C-15d, T-3d, and T-15d, respectively (Figure 3C).

### 2.4. ABA Treatment Affects the Expression of Genes Involved in Hormone Signaling Pathways

**Figure 4:**
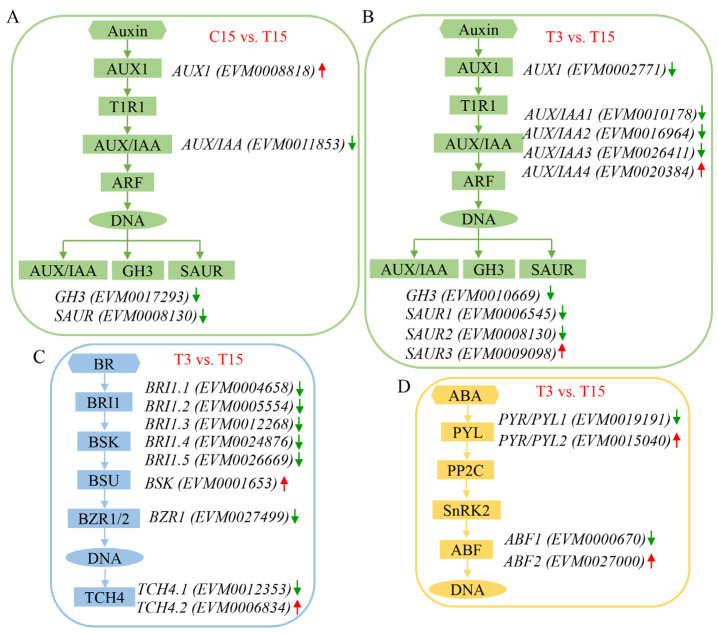
Analysis of plant hormone signal transduction during taro corm expansion. The red arrow on the right indicates an increase in gene expression, and the green arrow indicates a decrease. (**A**) DEGs related to auxin signal transduction in C15 vs. T15. (**B**) DEGs related to auxin signal transduction in T3 vs. T15. (**C**) DEGs related to brassinosteroid signal transduction in T3 vs. T15. (**D**) DEGs related to abscisic acid signal transduction in T3 vs. T15.

On the basis of KEGG pathway class analysis, DEGs were enriched mainly in the phenyl propanoid biosynthesis and plant hormone signal transduction pathways between C15 and T15. Four genes were identified in the auxin signaling pathway, one upregulated auxin influx carrier (*AUX1*) gene (EVM0008818), and three downregulated genes, namely, the auxin/indole-3-acetic acid (*AUX/IAA*) gene (EVM0011853), the gretchen hagen 3 (*GH3*) gene (EVM0017293), and the small auxin upregulated RNA (*SAUR*) gene (EVM0008130) (Figure 4A). In T3 vs. T15, the plant hormone signal transduction genes were enriched, and nine genes associated with the auxin signaling pathway were identified; these genes included a downregulated *AUX1* gene (EVM0002771) and four *AUX/IAA* genes, with three downregulated (EVM0010178, EVM0016964, and EVM0026411) and one upregulated gene (EVM0020384). Additionally, a downregulated *GH3* gene (EVM0010669) and three *SAUR* genes, with two downregulated (EVM0006545 and EVM0008130) and one upregulated (EVM0009098), were also identified (Figure 4B). In the brassinosteroid signaling pathway, five brassinosteroid insensitive 1 (*BRI1*) genes were downregulated (EVM0004658, EVM0005554, EVM0012268, EVM0024876, and EVM0026669), one brassinosteroid-signaling kinase (*BSK*) gene (EVM0001653) was upregulated, one brassinazole resistant 1 (*BZR1*) gene (EVM0027499) was downregulated, and two xyloglucan endotransglucosylase (*TCH4*) genes, with one downregulated (EVM0012353) and one upregulated (EVM0006834), were identified (Figure 4C). In the abscisic acid signaling pathway, two pyrabactin resistance/pyr1-like (*PYR/PYL*) genes were identified, where one was downregulated (EVM0019191) and one was upregulated (EVM0015040), and two ABRE binding factors (*ABF*) genes were identified, where one was downregulated (EVM0000670) and one was upregulated (EVM0027000) (Figure 4D).

### 2.5. Expression Patterns of Key Genes Associated with Taro Expansion

**Figure 5:**
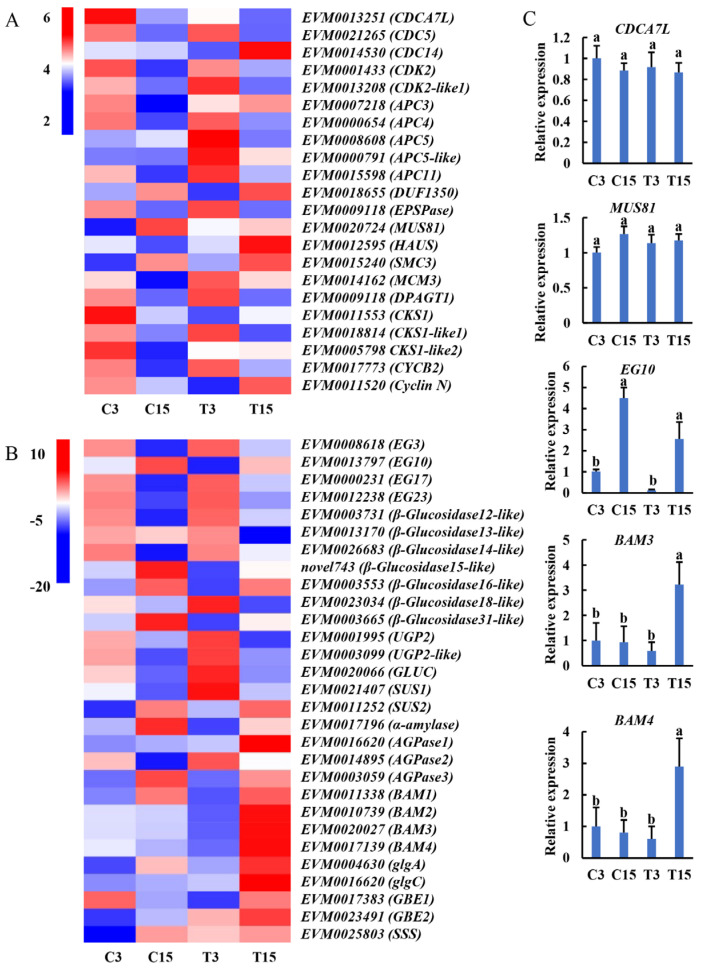
Expression patterns of key genes associated with taro corm expansion. (**A**) Genes related to cell division and the cell cycle: red and blue colors indicate upregulated and downregulated genes, respectively. (**B**) Genes related to starch and sucrose metabolism: red and blue colors indicate upregulated and downregulated genes, respectively. (**C**) Validation of the transcriptome data via qRT-PCR. The data are presented as the means ± SDs of three biological replicates. Different letters above the columns indicate a significant difference at *p* < 0.05 according to Duncan’s test.

Here, we identified genes related to the cell cycle, sugar transfer, and starch metabolism. In the process of taro expansion, genes related to cell division and cell cycle were highly expressed in C3 or T3 and gradually decreased in the later stage (Figure 5A). Previous studies have shown that in the early stage of tuber formation, many cells divide and proliferate [12]. Similarly, in the starch and sugar metabolism pathway, genes related to sucrose metabolism were expressed at C3 or T3, whereas genes related to starch synthesis were highly expressed at C15 or T15. Starch is the main form of starch found in tubers. In the process of tuber expansion, sucrose is converted into starch and accumulates in cells, resulting in tuber expansion. Moreover, the endoglucanase, glucosidase, *UGPase*, and *SUS* genes were upregulated in C3 vs. T3, whereas amylase, *AGPase*, β-amylase (*BAM*), the glycogen branching enzyme gene (*GBE*), and the soluble starch synthase gene (*SSS*) were highly expressed in C15 vs. T15 (Figure 5B). These results showed that 5 μM ABA induced taro tuber swelling and starch synthesis.

To verify the accuracy of the gene expression patterns, we selected five genes for qRT-PCR assays and found that the qRT-PCR results were consistent with the RNA-seq data (Figure 5C), indicating that the transcriptome data are convincing.

## 3. Discussion

### 3.1. The Shoot Apical Meristem Tissue Culture Is an Effective Method for Obtaining Virus-Free Taro

Taro is prone to virus accumulation during long-term asexual propagation, leading to progressive declines in quality and yield. DsMV, the most significant viral pathogen in the Araceae family [29], causes severe reductions in crop yield and quality upon infection [30]. Taro plants infected with DsMV exhibit stunted growth, reduced corm size, lower yields, and diminished quality [31]. Plant viruses typically invade host tissues via plasmodesmata and the vascular system. However, the shoot apical meristem, which consists primarily of undifferentiated meristematic cells lacking a mature vascular system and possessing incomplete plasmodesmata [32,33], serves as an effective tool for trait restoration and mitigating virus-induced losses. In this study, virus-free plantlets with stable genetic traits and low mutation rates were obtained through shoot apical meristem culture. RT-PCR assays confirmed the free DsMV in regenerated plants after 90 days of in vitro growth, demonstrating the successful production of virus-free taro shoots (Figure 1C). Plant viruses can induce phenotypic modifications in their hosts, often leading to the deterioration or loss of agriculturally desirable traits through disruptions in physiological processes. To preserve the favorable genetic traits of the germplasm and fully realize its potential for high yield and superior quality, the use of healthy, virus-free plant materials is essential [34]. This is of critical importance for the conservation of taro germplasm resources and the selection of new varieties.

The virus concentration decreases with proximity to the growth point in the shoot apical meristem (SAM), resulting in a lower probability of viral presence when smaller shoot apical meristem explants are used [32]. In this study, we observed stem tip differentiation, where explants formed greenish-yellow, firm-textured calli after approximately 20 d, followed by bud initiation at 30 d and adventitious shoot formation at 70 d. For taro SAM culturing, explants were maintained on an MS medium for four weeks before being transferred to an MS medium supplemented with either 2 mg/L BAP or 0.5 mg/L TDZ. After six weeks, the BAP and TDZ treatments yielded 2.1 and 2.18 adventitious shoots per explant, respectively [35].

### 3.2. High Concentrations of Sucrose and Exogenous ABA Are the Key Factors That Induce Taro Corm Expansion

Previous studies have shown that a high concentration of sucrose plays an important role in inducing and promoting the formation of storage organs in tuber crops. In cocoyam (*Xanthosoma sagittifolium* L. Schott), the shoot apical meristem was cultured in the MS medium supplemented with 3% sucrose, while the subsequent explants obtained were used for the production of microtubers on an MS-based plant growth regulator (PGR) free medium supplemented with 8% sucrose [36]. Higher concentrations of sucrose promoted microtuber production, microtuber size, and fresh weight, and increasing sucrose concentrations improved the in vitro microtuber production of potato without negative side effects [37]. In vitro production of microtubers in Chinese yam resulted in the highest microtuber index with 60 mg/L sucrose [38]. The sucrose concentration is one of the key factors affecting test-tube taro corm formation and expansion. In our study, 3% sucrose induced the formation of test-tube taro corms, whereas 8% sucrose significantly induced test-tube taro corm expansion (Figure 1).

In addition to participating in the stress response, ABA plays an important role in plant growth and development, especially in the development of tuber crops. In the early stage of storage root development, the ABA content increases significantly, and along with the upregulated expression of starch synthesis pathway-related genes, the starch content also significantly increases, ultimately promoting the formation of sweet potato tubers [39]. During the bulbous expansion of the gladiolus corm, ABA increased the content of sucrose and starch in the corm by upregulating the expression of *GhSUS2*, which positively regulated the development of the corm [15]. ABA promotes the reflux of nutrients from leaves to underground tubers, which accelerates the accumulation of starch and sugar and promotes the formation and development of tubers. In our previous study, an increase in ABA content during the expansion of taro corms was reported [16]. In this study, it was found that the TBM11 medium promoted taro corm expansion, while the taro corm only weighed 0.3 g in the TBM16 medium (Figure 1). Additionally, the taro plants withered after ABA treatment, suggesting that ABA facilitates the return of nutrients from the leaves to the taro corm and promotes corm expansion (Figure 2).

### 3.3. Working Model for Obtaining Virus-Free Taro Plantlets and Test-Tube Taro Corms via Shoot Apical Meristems

**Figure 6:**
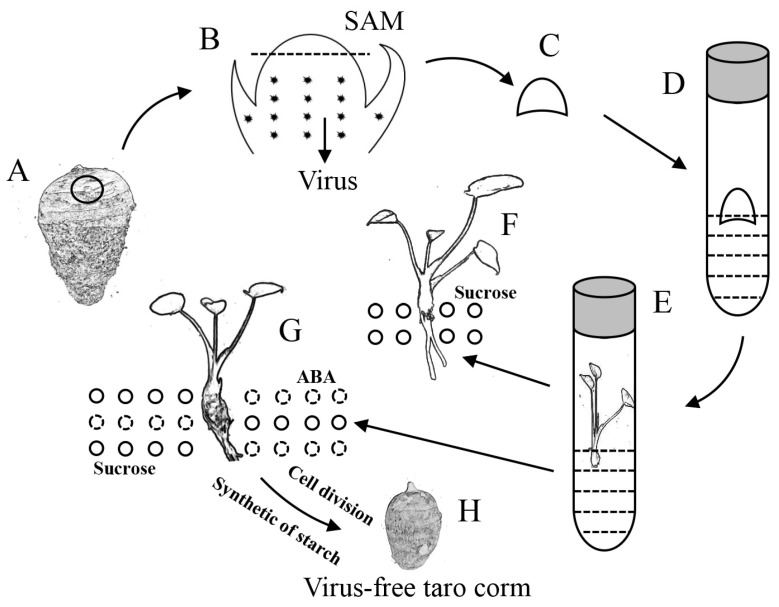
Model of virus-free taro plantlets and taro corms induced by sucrose and ABA. (**A**) Taro infected with the virus; the black circle represents the taro bud. (**B**) The shoot apical meristems from the taro bud; 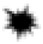 represents the taro virus, and the area above the dotted line was used to induce adventitious shoots. (**C**) The shoot apical meristems for inducing adventitious shoots. (**D**) The shoot apical meristem was cultured in vitro. (**E**) The shoot apical meristem was cultured into adventitious shoots, and an RT-PCR assay was used to verify the elimination effect. (**F**) Adventitious shoots only grow roots in 3% sucrose conditions, and the solid circles represent sucrose. (**G**) Adventitious shoots induced taro corms in 8% sucrose or 3% sucrose + 5 μM ABA conditions; the solid circles represent sucrose, and the dotted circles represent ABA. (**H**) The virus-free taro corm, which is easy to transport and store.

Virus-free taro plantlets were obtained through shoot apical meristem culture, and high concentrations of sucrose or low concentrations of sucrose combined with ABA were used to induce test-tube taro corms; subsequently, a working model was proposed (Figure 6A,B). By means of in vitro induction, the shoot apical meristem was induced into adventitious shoots in the culture medium (Figure 6C–E), and its effect on virus elimination was examined via RT-PCR. Adventitious shoots produced roots only in the presence of 3% sucrose (Figure 6F). Furthermore, adventitious shoots induced taro corms in the presence of 8% sucrose or 3% sucrose + 5 μM ABA (Figure 6G). Finally, virus-free taro corms, which are easy to transport and store, were obtained (Figure 6H). Collectively, our findings reveal a way to obtain virus-free taro, confirm that sucrose and ABA promote taro corm expansion in vitro, and identify some genes related to the cell cycle, starch synthesis, and metabolism.

## 4. Materials and Methods

### 4.1. Obtainment of Shoot Apical Meristems

Taro cultivars of ‘Wangen’ were used as in vitro research materials from Jiangxi Province Key Laboratory of Vegetable Cultivation and Utilization (Jiangxi Agricultural University). Taro mother plants infected with DsMV were previously identified and confirmed by the RT-PCR method prior to the experiments. The buds slightly protruding from the surface of the cormel were cut off with a scalpel and rinsed with running water for 30 min before being sterilized with a 1/1000 concentration of mercury dichloride (HgCl_2_) for 8 min. The buds were rinsed three times with sterile distilled water, sterilized with 75% alcohol for 30 s, and then rinsed three times with sterile distilled water. In a sterile environment, the taro leaf primordium was removed via a dissecting microscope until the shoot apical meristem unfolded, and the size of the shoot apical meristem was 0.2 mm, as previously described by Liu et al. [40].

### 4.2. Preparation of the Medium

In this study, a total of 16 different culture media were used to induce adventitious shoots, proliferate these shoots, and promote the expansion of taro corms (Table S2). Each liter of the culture medium was supplemented with 4.4 g of MS powder (PhytoTechnology Laboratories, M519, Lenexa, KS, USA) and 6.5 g of agar (Solarbio^®^, A8190, Beijing, China), and additional components as listed in Table S2. The pH of all media was adjusted to 5.8, and the media were sterilized at 121 °C and 103.4 kPa for 20 min. The medium was subsequently aliquoted into 200 mL (the diameter is 5.3 cm and the height is 9 cm) glass culture bottles or 5 mL (Biosharp^®^, BS-50-M, Hefei, China) tubes, with each bottle containing approximately 50 mL and each tube containing approximately 3.5 mL.

### 4.3. The Shoot Apical Meristem Culture

The 0.2 mm shoot apical meristems were cultured in 5 mL tubes with the TBM1–TBM3 medium; one shoot apical meristem was placed in each tube, and a total of 30 tubes were used for each treatment. The cultures were subsequently incubated in the growth room at 24 °C with a 16 h (light) and 8 h (dark) photoperiod, and the light intensity was 2000 Lux. The survival of the shoot apical meristems, which remained viable after cultivation, was determined after 30 d, and the surviving shoot apical meristems could grow into 1.5 cm adventitious shoots without subculture in 90 d.

### 4.4. RNA Extraction, RT-PCR Assays, and Taro Virus Detection

On the 90th day, RNA was extracted from the leaves of the test-tube taro shoots using a Quick RNA Kit (Promega, LS104, Madison, WI, USA) to detect elimination effects, and the field-grown plants, which were diagnosed as infected with DsMV after being planted into the field for 60 d, were used as positive controls. A 1 μg aliquot of total RNA from each sample was used for reverse transcription and first-strand cDNA synthesis using the PrimeScript™ RT reagent Kit (Takara, 6110A, San Jose, CA, USA). A 2×TSINGKE Master Mix (Tsingke, TSE004, Beijing, China) was used to perform RT-PCR analyses with three biological replicates and three technical replicates on a Thermal Cycler (Bio-Rad, T100, Hercules, CA, USA). The reaction conditions were 95 °C for 5 min, followed by 35 cycles of 95 °C for 30 s, 58 °C for 30 s, 72 °C for 30 s, and 72 °C for 5 min, and then stored at 4 °C. After RT-PCR, 1% agarose was used to detect the RT-PCR products, and the gene-specific primers used for RT-PCR were as follows: DsMV-F: GGGCTTGGGTGATGATGGA; DsMV-R: GCCTTTCAGTGTTCTCGCTTG [41].

### 4.5. Proliferation of Adventitious Shoots

The 1.5 cm adventitious shoots were cultured in the TBM4–TBM9 medium. Five adventitious shoots were each placed in 200 mL glass culture bottles, with a total of 45 bottles used for each treatment. The cultures were subsequently incubated in the growth room at 24 °C with a 16 h (light) and 8 h (dark) photoperiod, and the light intensity was 2000 Lux. The shoot proliferation rate was determined after 30 d, and 5 cm adventitious shoots were obtained in 45 d.

### 4.6. Induction of Corm Expansion in Taro

The virus-free adventitious shoots with a height of 5 cm were used to induce taro corm expansion in the TBM10–TBM16 medium. One or two adventitious shoots were placed in 200 mL glass culture bottles, with a total of 30 bottles used for each treatment. Then, the cultures were incubated in the growth room at 24 °C with a 16 h (light) and 8 h (dark) photoperiod, and the light intensity was 2000 Lux. After 8 d of growth, the adventitious shoots were transferred to the same media described above, but in the absence of ABA or NDGA. The weight, transverse diameter, and longitudinal diameter of the test-tube taro corm were measured at 90 d.

### 4.7. RNA-Seq and Bioinformatics Analysis

On the 3rd and 15th days, the medium-contacted taro was collected, which had been in the TBM13 (Control, C) and TBM14 (Treatment, T) media for RNA-sequencing (RNA-seq) analysis, and they were re-named C-3, C-15, T-3, and T-15, respectively. Three biological replicates of RNA-seq libraries were constructed and sequenced using the Illumina HiSeq^TM^ 2500 platform (Illumina, San Diego, CA, USA) at Benagene Company (Wuhan, China). The clean reads were aligned to the taro reference genome, and a base quality test was conducted via SOAPnuke (version 2.0) and Trimmomatic (version 0.39) software [42]. Differential expression analysis (fold change ≥ 2, false discovery rate < 0.01) was performed with DESeq2 (version 1.40) software [43]. All DEGs were subjected to Kyoto Encyclopedia of Genes and Genomes (KEGG) analysis (http://www.kegg.jp/, accessed on 1 February 2025), and principal component analysis (PCA) was performed using the prcomp function in TBtools (version 2.056) software [44].

### 4.8. Validation of the Transcriptome Data via qRT-PCR Assay

To verify the correctness of the transcriptome data, a total of 5 genes were selected for quantitative real-time polymerase chain reaction (qRT-PCR) analysis. One microgram of total RNA was used to synthesize cDNA with the PrimeScript™ RT reagent Kit (Takara, Japan), and the qRT-PCR assay was performed on a Roche LightCycler 480 system using TB Green^®^ Premix Ex Taq™ (Takara, Japan). Each reaction mixture contained 10 ng cDNA, 0.5 µM forward primer (primer-F), 0.5 µM reverse primer (primer-R), 10 µL TB Green Mix, and ddH₂O for a final volume of 20 µL, with three replicates included for each sample. The cycling conditions were as follows: initial denaturation at 95 °C for 5 min, followed by 40 cycles of 95 °C for 15 s, 60 °C for 30 s, and 72 °C for 30 s. Actin (Unigene11572_All) was used as the internal reference gene to normalize the expression data [16], and the 2^−ΔΔCT^ method was used to calculate the relative expression levels [45]. The primers used are listed in Table S3.

## 5. Conclusions

DsMV-free test-tube taro corms can be obtained by shoot apical meristem culture, with 93.3% of the test-tube shoots being virus-free. Sucrose (8%) can significantly promote taro corm expansion, and 3% sucrose +5 μM ABA can induce taro corm. Transcriptome data revealed that genes related to cell division and the cell cycle, such as *CDK*, *CKS*, and *CYCB2*, were highly expressed during taro expansion. Sucrose and starch metabolism pathway genes, such as *SUS*, *BAM*, *GBE*, and *SSS*, were significantly enriched. Additionally, a working model was proposed based on the induction of test-tube taro corms using either high-concentration sucrose or a combination of low-concentration sucrose + ABA.

## Figures and Tables

**Table 1:** Growth status of test-tube taro under different media in 90 d.

Medium	Corm Weight (g)	Corm Transverse Diameter (mm)	Corm Longitudinal Diameter (mm)
TBM10	-	-	-
TBM11	0.40 ± 0.04 c	8.41 ± 0.26 b	9.64 ± 0.78 ab
TBM12	0.28 ± 0.03 d	7.23 ± 0.43 c	9.26 ± 0.99 b
TBM13	0.50 ± 0.01 b	8.79 ± 0.30 ab	11.85 ± 0.93 a
TBM14	0.62 ± 0.05 a	9.72 ± 0.54 a	11.98 ± 1.00 a
TBM15	0.26 ± 0.02 d	6.79 ± 0.40 c	8.80 ± 0.33 b
TBM16	0.30 ± 0.03 d	6.65 ± 0.30 c	8.01 ± 0.95 b

Note: Data are represented as the mean ± SD of three biological replicates. Different letters indicate a significant difference at *p* < 0.05 by Duncan’s test.

## Data Availability

All data are displayed in the manuscript and Supplementary Materials.

## Supplementary Materials

The following supporting information can be downloaded at: https://www.mdpi.com/article/10.3390/ijms26083740/s1.
