# Supplementary material for: Virus-Free Micro-Corm Induction and the Mechanism of Corm Development in Taro
Source: Int J Mol Sci. 2025 Apr 16;26(8):3740. doi: 10.3390/ijms26083740 (PMC12027564; doi:10.3390/ijms26083740)
Supplement: Supplementary file 1 [file ijms-26-03740-s001.zip › ijms-3519946-supplementary.pdf]

Table S1. Quality statistics of clean reads in 12 libraries.

Sample	Raw Reads (M)	Clean Reads (M)	Clean Bases (Gb)	Clean Reads Q20 (%)	Clean Reads Q30 (%)	GC content (%)	Total reads(bp)	Unique mapped(%)
C3-1	41.38	41.32	5.99	97	92	50	21,664,278	84.36
C3-2	41.36	41.29	5.97	97	92	50	21,647,995	83.47
C3-3	41.38	41.31	5.98	97	91	50	21,660,423	84.9
C15-1	41.38	41.33	6.00	97	92	51	21,666,730	82.62
C15-2	41.34	41.27	6.00	97	92	50	21,639,183	84.05
C15-3	41.35	41.29	5.99	97	92	51	21,646,719	83.1
T3-1	41.37	41.31	5.99	97	92	50	21,656,807	85.34
T3-2	41.33	41.28	5.99	97	91	50	21,641,897	84.92
T3-3	41.35	41.29	5.98	97	92	50	21,648,896	85.45
T15-1	41.39	41.33	6.00	97	92	50	21,667,908	83.87
T15-2	41.38	41.32	6.02	97	91	50	21,664,903	83.35
T15-3	41.38	41.31	6.00	97	92	50	21,658,202	83.5
average	41.37	41.30	5.99	97	92	50	21,6553,28	84.08

Table S2. The additional components of the culture medium.

Media	Sucrose (g/L)	6-BA (mg/L)	NAA (mg/L)	ABA (mg/L)	NDGA (mg/L)
TBM1	30	0.5	0.1	0	0
TBM2	30	1	0.3	0	0
TBM3	30	2	0.5	0	0
TBM4	30	1	0.1	0	0
TBM5	30	2	0.2	0	0
TBM6	30	3	0.5	0	0
TBM7	30	1.5	0.3	0	0
TBM8	30	1	0.3	0	0
TBM9	30	1.5	0.5	0	0
TBM10	30	0	0	0	0
TBM11	30	0	0	1.32	0
TBM12	30	0	0	2.64	0
TBM13	80	0	0	0	0
TBM14	80	0	0	1.32	0
TBM15	80	0	0	2.64	0
TBM16	80	0	0	0	3.02

Table S3. Primers for qRT-PCR assays.

	F (5′→3′)	R (5′→3′)
CDCA7L	ACGTTTCCCTTGCTTCCGAT	TCCTACAGAAGATCCGGCCA
MUS81	CAAGGGGTTTTCCCGAAGGA	GGCAATTCCCCTGGAACAGA
EG10	GAGCCTGCTGGGATCTTTGT	AGAACTGCAGAGCATGGCTT
BAM3	TCACCCTGAGTGGTCATTGCCA	TGCGTGGTTGGGCTCTTTAT
BAM4	TGTCAGGGTTCGCTCACAAA	AACCAGCACTCAGCACCTAC
